# Light Effect on Water Viscosity: Implication for ATP Biosynthesis

**DOI:** 10.1038/srep12029

**Published:** 2015-07-08

**Authors:** Andrei P. Sommer, Mike Kh. Haddad, Hans-Jörg Fecht

**Affiliations:** 1 Institute of Micro and Nanomaterials, University of Ulm, 89081 Ulm, Germany

## Abstract

Previous work assumed that ATP synthase, the smallest known rotary motor in nature, operates at 100% efficiency. Calculations which arrive to this result assume that the water viscosity inside mitochondria is constant and corresponds to that of bulk water. In our opinion this assumption is not satisfactory for two reasons: (1) There is evidence that the water in mitochondria prevails to 100% as interfacial water. (2) Laboratory experiments which explore the properties of interfacial water suggest viscosities which exceed those of bulk water, specifically at hydrophilic interfaces. Here, we wish to suggest a physicochemical mechanism which assumes intramitochondrial water viscosity gradients and consistently explains two cellular responses: The decrease and increase in ATP synthesis in response to reactive oxygen species and non-destructive levels of near-infrared (NIR) laser light, respectively. The mechanism is derived from the results of a new experimental method, which combines the technique of nanoindentation with the modulation of interfacial water layers by laser irradiation. Results, including the elucidation of the principle of light-induced ATP production, are expected to have broad implications in all fields of medicine.

There is no other constituent of the cell which has fascinated both the life-science and the nanoscience community as much as the mitochondrial rotary motor, called ATP synthase. Initially it was estimated that the nanomotor operates at 100% efficiency^1^,^2^,^3^,^4^ — a picture based on several idealized assumptions, including that the viscosity of the milieu surrounding the motor is that of bulk water^5^,^6^,^7^,^8^,^9^, thus, paying no attention to viscosity gradients near surfaces^10^. Ignoring this point is critical because the mechanical behavior of molecular machines is different from that of their macroscopic analogues and many macroscopic mechanical concepts no longer apply at the molecular level. This concerns particularly the application of the concept of viscous friction and lubrication^11^,^12^. Recent experimental work showed that it is important to discriminate between the physical properties of bulk water and those of the nanoscopic interfacial water layers, which are masking surfaces. Nanoscopic water layers bound to hydrophilic surfaces (bound water) present viscosity values which are orders of magnitude larger than those of bulk water^13^. Furthermore, it was experimentally shown that with increasing confinement between hydrophilic surfaces, the viscosity of nanoscopic water layers dramatically increases^14^,^15^.

In an intuitive attempt to extend the aforementioned findings to the mitochondrial rotary motor, we emphasize that it consists of a hydrophobic and a predominantly hydrophilic part F_0_ and F_1_, respectively^16^. Inevitably, components of the nanomotor will operate proximal to hydrophilic surfaces and in subnanometer gaps. Local variations in viscosity are expected to play a considerable role, specifically near the hydrophilic moiety (F_1_) and presumably in the contact zone between F_1_ and F_0_ where viscous friction probably affects the dynamics and the efficiency of the nanomotor system. Considering that the classical imaging tools employed to investigate the structure of the mitochondrial nanomotor — scanning electron microscope and transmission electron microscope — operate in vacuum, it is clear that any relevant information related to nanoscopic interfacial water layers, which are masking the surface of the nanomotor or are captured between its molecular constituents, is lost^17^. In addition, it should be also stressed that the nanomotor operates in the interior of mitochondria: a crowded, predominantly hydrophilic and highly viscous microenvironment in which the fraction of water prevalent as bound water has been estimated to approach 100 per cent of the total water content^18^. Consideration of this experimental result would already be sufficient to challenge the utilization of the viscosity of bulk water in the models and simulations used to assess the efficiency of the mitochondrial nanomotor.

The principal function of the mitochondrial ATP synthase is the synthesis of ATP — the primary energy carrier in cells. The root cause of the synthesis is the translocation of protons, which are turning the rotor^19^,^20^. The protons are driven across the inner membrane of mitochondria into the mitochondrial matrix by the energy of the proton gradient, the transmembrane proton-motive force^21^. It is thus plausible to assume that processes with direct impact on the dynamics of the nanomotor will affect ATP production. For instance, reactive oxygen species (ROS) induced oxidative stress was shown to cause depletion of ATP levels in mitochondria^22^,^23^, apparently a mechanism, which works in both mammalian and plant cells^24^. The question arises: What is the intrinsic mechanism by which ATP is depleted by elevated levels of ROS? Coincidentally, both compounds ATP and ROS are produced in mitochondria. Therefore, from the premise that bursts of ROS will accentuate the hydrophilic nature of the intramitochondrial space (due to the oxygen), the most plausible answer is that ROS enhances hydrophilicity, and thereby the viscous friction between surfaces moving relative to each other. Hence, pathological conditions that are triggering prolonged bursts of ROS will contribute to a transient increase in the viscosity of the interfacial water layers bound to exposed intramitochondrial surfaces. An increase in interfacial viscosity, concomitant with an increase in viscous friction, can only manifest itself in a reduced performance of the rotary motor, which explains the drop in ATP production. Indeed, molecular dynamics simulations predicted that interfacial viscosity would increase with hydrophilicity^25^. Recently, the correlation between viscosity and hydrophilicity received further confirmation by atomic force microscopy: The measured interfacial viscous forces were larger for materials with smaller contact angles, i.e., more hydrophilic surfaces, and vice versa^15^.

If indeed, a raise in interfacial viscosity impacts the rotation of the mitochondrial nanomotor, for instance, under conditions of prolonged oxidative stress, which are inescapably present during *in vitro* experiments, it can be reasonably assumed that a reduction of potentially elevated viscosity levels will manifest itself in an increase in ATP production. Earlier, we showed that the structure of nanoscopic interfacial water layers (about 2–3 monolayers)^26^ can be modulated with 670 nm laser light applied at moderate intensities as low as 50 W ∙ m^−2^^27^. Modulation includes volume expansion^28^ and viscosity reduction, specifically on hydrophilic surfaces, which are known to promote high viscosities^14^. Importantly, modulation effects were not restricted to 670 nm light. Comparable modulation effects were realized with other laser wavelengths, for instance, 633 nm, applied at 400 W ∙ m^−2^^26^. The expansion effect was exploited *in vitro* to force cancer cells to uptake various cytostatic drugs^29^, and predicted to be instrumental in the release of drugs from permeable nanovesicles, a prediction that has recently been confirmed experimentally^30^. Summarizing, there is observational evidence for the prevalence of intramitochondrial water viscosity gradients, and for a decrease and increase in ATP synthesis in response to ROS and laser light^31^, respectively. If indeed the root cause of the variation in ATP production is a variability in intramitochondrial viscous friction, then the same light which stimulated ATP production in cells should also reduce the viscous friction in subnanometer gaps.

## Results and Discussion

### Probing nanoscopic interfacial water layers by nanoindentation and NIR laser light

**Figure 1:**
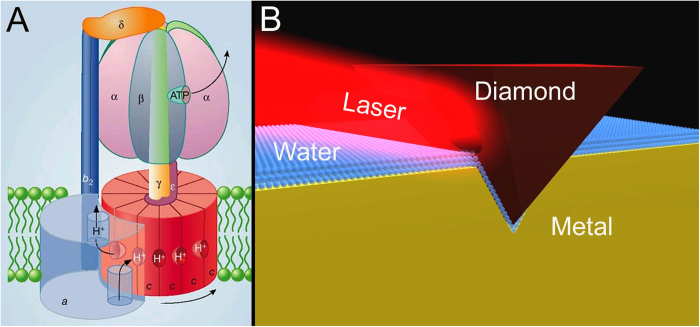
Mitochondrial nanomotor (**A**). During ATP synthesis, the rotor turns about 9000 times per minute. Artists view of the principle of light-tuned nanoindentation. Blue spheres stand for water molecules forming the nanoscopic water layers confined in the space between the diamond tip and nanoindentation imprint (**B**). Reprinted by permission from Macmillan Publishers Ltd: [NATURE]^39^, copyright (2004).

**Figure 2:**
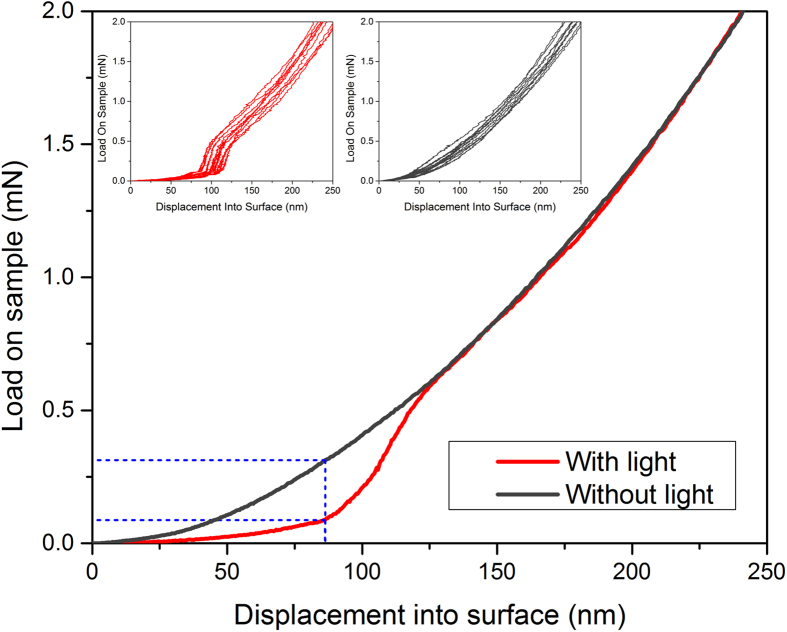
Nanoindentation loading curves (average curves, n = 10) for hydrophilic sample (single crystal gold), with and without 670 nm laser. The force required for the diamond tip to penetrate ~100 nm into the metal is less when the tip/substrate contact zone is irradiated. Insets display the corresponding measurements shown as individual curves. Representative curves for single crystal aluminum have been presented during the International Conference on Laser Applications in Life Sciences, 2014^40^.

Here, we report on laboratory experiments focusing on nanoscopic interfacial water layers which prevail on hydrophilic surfaces and are confined in subnanometer gaps, and their light tunability. Similar water layers are expected to determine the efficiency of the rotary nanomotor (Fig. 1A). For their exploration we used a nanoindenter. We recorded the force required to penetrate 1 μm into different model substrates. We probed both hydrophilic and hydrophobic substrate materials, in dark and during irradiation of the tip/substrate contact zone with 670 nm laser light. Figure 1B illustrates the principle of the experiment. Hydrophilic materials comprised aluminum, zinc, copper and gold, while polymers, for instance, polystyrene, served as hydrophobic materials. Figure 2 presents the mean value curves of 2 × 10 representative nanoindentation measurements performed on a hydrophilic substrate (single crystal gold), irradiated with 670 nm laser light, and without laser irradiation. In both cases the measurements were performed in a closed box at room temperature and constant relative humidity of 67%. As can be clearly seen in Fig. 2 the laser light contributed to a reduction in the load required to penetrate the hydrophilic sample (assumed to carry a viscous film of interfacial water which is tunable by 670 nm laser light via collective interaction of photons with transiently immobilized interfacial water molecules)^26^,^27^,^28^. The drop in the load with laser light was estimated to be around 72% compared to the non-irradiated sample. The results for all tested metals are summarized in Table 1. Furthermore, at a relative humidity threshold of 48%, the effect disappeared, indicating that the amount of water confined at the tip/cavity interface was too small to build up a substantial viscous layer, reflecting a correlation with environmental humidity. On the basis of the humidity dependence we can safely exclude that the cause of the observed effect was the heating of the diamond tip and/or substrate. Notably, when we probed the hydrophobic species, the laser light had practically no effect. The results are in agreement with the observational evidence that elevated interfacial viscosity levels are present on hydrophilic, but not on hydrophobic surfaces. If we consider the classical friction components (adhesion, ploughing and asperity deformation) as well as the localized plastic deformation which presumably occur during the penetration of the diamond tip into the substrates, it is obvious that none of these factors can be modified by the low intensity laser light. Thus, it seems reasonable to assume that the origin of the relatively low force required to penetrate the first 100 nanometers into the hydrophilic samples, as depicted in Fig. 2, is a reduction of the viscous friction in the tip/cavity interface by the laser light. Going back to mitochondria, it is now tempting to assume that the nanomotor efficiency (ATP productivity) can be tuned with biologically tolerated intensities of red to NIR laser light. This perspective receives justification from the experimental side: Previously, it was reported that red laser light (632.8 nm (power 15 mW, fluence 5 J ∙ cm^−2^) changed the energy metabolism in mitochondria irradiated *in vitro*, and caused an increase in ATP synthesis. It was suggested that the extra ATP synthesis is directly produced by a laser-induced extra proton-motive force^31^. Remarkably, comparable levels of 670 nm laser light (power 33 mW, fluence 1 J ∙ cm^−2^) increased both the proliferation and ATP production of cells *in vitro*^32^. We believe that the cause which gives the extra ATP is a reduction of interfacial viscosity within and/or around the mitochondrial nanomotor. The high torque of the nanomotor is best illustrated by its capacity to rotate objects several hundred times as large as the motor itself against the viscous friction of water. Torque is transferred to the ATP-producing part by the unit γ (Fig. 1). It is instructive to compare the mitochondrial rotary motor with a rotating cylinder viscometer. The measuring principle of the latter is based on the proportionality between the viscosity (η) and torque (T) 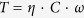, where C is a constant specific to the instrument and ω stands for the angular velocity of the rotating cylinder^33^. A reduction in viscosity by the laser light will increase the efficiency of the nanomotor, reflected by an increase in ATP production^31^.

### NIR laser light upregulates ATP synthesis

Interestingly, equal levels of red to near-infrared (NIR) light are used routinely in clinical praxis to accelerate the healing of complicated wounds^34^, to treat pain and inflammatory processes^35^. Coincidentally, the therapeutic use of NIR light plays an increasingly important role in aeronautic medicine. As the gravitational force increases or decreases, the cell function responds in a linear fashion. This poses significant health risks for astronauts in long-term spaceflight. The use of NASA LEDs (central wavelengths 670 nm) will significantly improve the medical care that is available to astronauts on long-term space missions^36^. For the majority of biostimulatory effects of NIR light described in the literature^37^, irradiation parameters were virtually identical to those found to reduce the viscosity of interfacial water layers. Apparently, irradiation with biologically tolerated levels of laser light shows pronounced effects in biological systems which are exposed to oxidative stress. Thus, we feel justified to assume that the irradiation upregulates ATP turnover by reducing the viscosity of the nanoscopic interfacial water layers which seem to control the efficiency of the mitochondrial nanomotor. The insight deduced from our laboratory experiments is expected to allow the improvement of the present theories and hypotheses of light-induced ATP synthesis, and promises to enhance the predictive capability of existing models. Explicitly, realistic models designed to explore the functioning of ATP synthase may have to consider interfacial viscosity gradients, within and around the nanoturbine. This aspect is of considerable biological interest and may lead to a shift in the paradigm of ATP synthesis.

## Methods

### Nanoindentation measurements and data analysis

A nanoindenter (Nanoindenter XP, Nano Instruments Inc. Oak Ridge, Tennessee, U.S.A.) equipped with a Berkovich tip (three-faceted diamond pyramid with a total included tip angle of 130.6° and tip radius <20 nm) was used to measure the load required for the tip to penetrate into the surface of different model substrates (hydrophilic and hydrophobic materials) to a depth of 1 μm through a complete load/unload cycle. Data analysis focused on penetration depths of a few 100 nm. Hydrophilic materials comprised single crystal aluminum, single crystal gold, zinc and copper; polymers, for instance, polystyrene, served as hydrophobic materials.

### Laser irradiation

Substrates were explored by nanoindentation in dark and during irradiation of the tip/substrate contact zone with 670 nm laser light (power 4.5 mW, intensity 4.0 kW ∙ m^−2^). Details of the laser configuration were reported previously^38^. The measurements were performed in a closed box at room temperature and constant relative humidity of 67%. At relative humidity levels below 48% the effect disappeared, in accordance with analogous measurements performed by atomic force acoustic microscopy^27^.

## Additional Information

**How to cite this article**: Sommer, A. P. *et al.* Light Effect on Water Viscosity: Implication for ATP Biosynthesis. *Sci. Rep.* **5**, 12029; doi: 10.1038/srep12029 (2015).

## Figures and Tables

**Table 1:** Reduction of indentation load in response to 670 nm laser irradiation.

Metal	Au	Al	Zn	Cu
Drop in load [%]	72	56	70	80

## References

[b1] Yasuda R., Noji H., Kinosita K. Jr. & Yoshida M. F1-ATPase is a highly efficient molecular motor that rotates with discrete 120° steps. Cell 93, 1117–1124 (1998). 9657145 10.1016/s0092-8674(00)81456-7

[b2] Saraste M. Oxidative phosphorylation at the *fin de siècle*. Science 283, 1488–1493 (1999). 10066163 10.1126/science.283.5407.1488

[b3] Kinosita K. Jr., Yasuda R., Noji H. & Adachi K. A rotary molecular motor that can work at near 100% efficiency. Philos. Trans. R. Soc. Lond. B Biol. Sci. 355, 473–489 (2000). 10836501 10.1098/rstb.2000.0589 PMC1692765

[b4] Toyabe S., Watanabe-Nakayama T., Okamoto T., Kudo S. & Muneyuki E. Thermodynamic efficiency and mechanochemical coupling of F1-ATPase. Proc. Natl. Acad. Sci. U. S. A. 108, 17951–17956 (2011). 21997211 10.1073/pnas.1106787108 PMC3207686

[b5] Kinosita K. Jr. Real time imaging of rotating molecular machines. FASEB J. 13, Suppl. 2, S201–S208 (1999). 10619128 10.1096/fasebj.13.9002.s201

[b6] Xu L. The coupled chemomechanics of the F1-ATPase molecular motor. Biochim. Biophys. Acta. 1777, 1422–1431 (2008). 18823935 10.1016/j.bbabio.2008.08.010

[b7] Usukura E. *et al.* Torque generation and utilization in motor enzyme F0F1-ATP synthase. J. Biol. Chem. 287, 1884–1891 (2012). 22128167 10.1074/jbc.M111.305938 PMC3265869

[b8] Zimmermann E. & Seifert U. Efficiencies of a molecular motor: A generic hybrid model applied to the F1-ATPase. New J. Phys. 14, 103023 (2012).

[b9] Junge W. Bioenergetics in mitochondria, bacteria and chloroplasts: Half a century of molecular bioenergetics. Biochem. Soc. Trans. 41, 1207–1218 (2013). 24059510 10.1042/BST20130199

[b10] Chiwata R. *et al.* None of the rotor residues of F1-ATPase are essential for torque generation. Biophys. J. 106, 2166–2174 (2014). 24853745 10.1016/j.bpj.2014.04.013 PMC4052266

[b11] Vogelsberg C. S. & Garcia-Garibay M. A. Crystalline molecular machines: function, phase order, dimensionality, and composition. Chem. Soc. Rev. 41, 1892–1910 (2012). 22012174 10.1039/c1cs15197e

[b12] Panman M. R. *et al.* Water lubricates hydrogen-bonded molecular machines. Nat. Chem. 5, 929–934 (2013). 24153370 10.1038/nchem.1744

[b13] Jinesh K. B. & Frenken J. W. Capillary condensation in atomic scale friction: How water acts like a glue. Phys. Rev. Lett. 96, 166103 (2006). 16712250 10.1103/PhysRevLett.96.166103

[b14] Goertz M. P., Houston J. E. & Zhu X. Y. Hydrophilicity and the viscosity of interfacial water. Langmuir 23, 5491–5497 (2007). 17408290 10.1021/la062299q

[b15] Ortiz-Young D., Chiu H. C., Kim S., Voïtchovsky K. & Riedo E. The interplay between apparent viscosity and wettability in nanoconfined water. Nat. Commun. 4, 2482 (2013). 24052015 10.1038/ncomms3482

[b16] Hutcheon M. L., Duncan T. M., Ngai H. & Cross R. L. Energy-driven subunit rotation at the interface between subunit a and the c oligomer in the F0 sector of *Escherichia coli* ATP synthase. Proc. Natl. Acad. Sci. U. S. A. 98, 8519–8524 (2001). 11438702 10.1073/pnas.151236798 PMC37468

[b17] Minauro-Sanmiguel F., Wilkens S. & García J. J. Structure of dimeric mitochondrial ATP synthase: Novel F0 bridging features and the structural basis of mitochondrial cristae biogenesis. Proc. Natl. Acad. Sci. U. S. A. 102, 12356–12358 (2005). 16105947 10.1073/pnas.0503893102 PMC1194923

[b18] Ford R. C. *et al.* Inelastic incoherent neutron scattering measurements of intact cells and tissues and detection of interfacial water. J. Am. Chem. Soc. 126, 4682–4688 (2004). 15070386 10.1021/ja0393269

[b19] Nagyvary J. & Bechert J. New insights into ATP synthesis. Biochem. Educ. 27, 193–199 (1999).

[b20] Watt I. N., Montgomery M. G., Runswick M. J., Leslie A. G. & Walker J. E. Bioenergetic cost of making an adenosine triphosphate molecule in animal mitochondria. Proc. Natl. Acad. Sci. U. S. A. 107, 16823–16827 (2010). 20847295 10.1073/pnas.1011099107 PMC2947889

[b21] Mitchell P. Coupling of phosphorylation to electron and hydrogen transfer by a chemi-osmotic type of mechanism. Nature 191, 144–148 (1961). 13771349 10.1038/191144a0

[b22] Janero D. R., Hreniuk D. & Sharif H. M. Hydroperoxide-induced oxidative stress impairs heart muscle cell carbohydrate metabolism. Am. J. Physiol. 266, C179–C188 (1994). 8304415 10.1152/ajpcell.1994.266.1.C179

[b23] Chen H. *et al.* NAD^+^-carrying mesoporous silica nanoparticles can prevent oxidative stress-induced energy failures of both rodent astrocytes and PC12 cells. PLoS One 8, e74100 (2013). 24040179 10.1371/journal.pone.0074100 PMC3767595

[b24] Tiwari B. S., Belenghi B. & Levine A. Oxidative stress increased respiration and generation of reactive oxygen species, resulting in ATP depletion, opening of mitochondrial permeability transition, and programmed cell death. Plant Physiol. 128, 1271–1281 (2002). 11950976 10.1104/pp.010999 PMC154255

[b25] Sendner C., Horinek D., Bocquet L. & Netz R. R. Interfacial water at hydrophobic and hydrophilic surfaces: Slip, viscosity, and diffusion. Langmuir 25, 10768–10781 (2009). 19591481 10.1021/la901314b

[b26] Sommer A. P., Zhu D., Försterling H.-D., Scharnweber T. & Welle A. Crystalline water at room temperature − Under water and in air. Cryst. Growth Des. 8, 2620–2622 (2008).

[b27] Sommer A. P., Caron A. & Fecht H.-J. Tuning nanoscopic water layers on hydrophobic and hydrophilic surfaces with laser light. Langmuir 24, 635–636 (2008). 18171094 10.1021/la7032737

[b28] Sommer A. P. *et al.* Breathing volume into interfacial water with laser light. J. Phys. Chem. Lett. 2, 562–565 (2011).

[b29] Sommer A. P., Zhu D. & Scharnweber T. Laser modulated transmembrane convection: Implementation in cancer chemotherapy. J. Control Release 148, 131–134 (2010). 20934473 10.1016/j.jconrel.2010.10.010

[b30] Carter K. A. *et al.* Porphyrin-phospholipid liposomes permeabilized by near-infrared light. Nat. Commun. 5, 3546 (2014). 24699423 10.1038/ncomms4546 PMC3988818

[b31] Passarella S. *et al.* Increase of proton electrochemical potential and ATP synthesis in rat liver mitochondria irradiated *in vitro* by helium-neon laser. FEBS Lett. 175, 95–99 (1984). 6479342 10.1016/0014-5793(84)80577-3

[b32] Sommer A. P. *et al.* 670 nm laser light and EGCG complementarily reduce amyloid-β aggregates in human neuroblastoma cells: Basis for treatment of Alzheimer’s disease? Photomed. Laser Surg. 30, 54–60 (2012). 22029866 10.1089/pho.2011.3073

[b33] Viswanath D. S., Ghosh T. K., Prasad D. H. L., Dutt N. V. K. & Rani K. Y. Viscosity of Liquids: Theory, Estimation, Experiment, and Data. Springer; pp 61–63 (2007).

[b34] Mester E., Mester A. F. & Mester A. The biomedical effects of laser application. Lasers Surg. Med. 5, 31–39 (1985). 3982191 10.1002/lsm.1900050105

[b35] Mester A. Laser biostimulation. *Photomed. Laser Surg*. 31, 237–239 (2013). 23741992 10.1089/pho.2013.9876

[b36] Whelan H. T. The use of NASA light-emitting diode near-infrared technology for biostimulation. In *Proceedings of the 2*^*nd*^ *Int. Conf. on NOA*, Johnson Space Center, May 2001, Houston/TX, USA; NASA Publication CP-2002-210786 (2002).

[b37] Sommer A. P., Pinheiro A. L. B., Mester A. R., Franke R. P. & Whelan H. T. Biostimulatory windows in low-intensity laser activation: lasers, scanners, and NASA’s light-emitting diode array system. J. Clin. Laser Med. Surg. 19, 29–33 (2001). 11547815 10.1089/104454701750066910

[b38] Sommer A. P. *et al.* Interfacial water an exceptional biolubricant. Cryst. Growth Des. 9, 3852–3854 (2009).

[b39] Cross R. L. Turning the ATP motor. Nature, 427, 407–408 (2004). 14749816 10.1038/427407b

[b40] Sommer A. P., Haddad M. K. h. & Fecht H. J. Tuning the wheel of life with light. In Abstracts of the International Conference on Laser Applications in Life Sciences, Ulm, Germany, June 29 – July 2 (2014).

